# Unusual Cases of Monoclonal Gammopathy of Renal Significance

**DOI:** 10.1155/2024/5556426

**Published:** 2024-09-12

**Authors:** Anjellica Chen, Anna-Ève Turcotte, Sarah Higgins, Michel Pavic, Vincent Ethier, Vincent Lévesque Dion

**Affiliations:** ^1^ Department of Internal Medicine Université de Sherbrooke, Sherbrooke, Canada; ^2^ Department of Nephrology Centre Hospitalier Universitaire de Sherbrooke Université de Sherbrooke, Sherbrooke, Canada; ^3^ Department of Hematology Centre Hospitalier Universitaire de Sherbrooke Université de Sherbrooke, Sherbrooke, Canada; ^4^ Department of Pathology CIUSSS de la Mauricie-et-du-Centre-du-Québec, Trois-Rivières, Canada

## Abstract

**Introduction:**

Monoclonal gammopathy of renal significance (MGRS) is a rare entity describing patients with renal impairment related to the secretion of immunoglobulins without hematological criteria for treatment of a specific disease. We present 3 cases of MGRS identified at our center that were either rare or difficult to diagnose. *Case Presentations*. The first patient presented with monoclonal membranoproliferative glomerulonephritis in the context of known chronic lymphocytic leukemia (CLL), diagnosed about 10 years prior. She presented with nephritic syndrome with serum protein electrophoresis revealing an IgG/lambda peak of less than 1 g/L, stable from the last few years. A renal biopsy confirmed a diagnosis of monoclonal membranoproliferative glomerulonephritis with granular IgG and C3 deposits of various sizes. The second patient presented with renal TMA in the context of IgM MGUS. The patient was admitted for acute nephritic syndrome and thrombotic microangiopathy. Serum protein electrophoresis demonstrated IgM/kappa paraprotein at 1.8 g/L, with a kappa/lambda ratio of 5.48. Renal biopsy demonstrated endocapillary proliferative glomerulonephritis associated with the presence of numerous monotypic IgM/kappa intracapillary pseudothrombi. Characteristic changes of thrombotic microangiopathy were also described. The third patient presented with immunotactoid glomerulonephritis likely from small B-cell lymphoma that later transformed to DLBCL. The patient presented with acute renal failure with IgM/kappa paraprotein of less than 1 g/L on electrophoresis and with a kappa/lambda ratio of 7.09. A diagnosis of immunotactoid glomerulonephritis was made on renal biopsy. Bone marrow with limited specimen revealed a B-cell infiltrate. Biopsy of a breast lesion was compatible with diffuse large B-cell lymphoma (DLBCL). Lymphomatous cells expressed IgM/kappa, thus confirming paraprotein-associated renal lesion.

**Conclusion:**

We described 3 different cases of MGRS, highlighting the diversity of renal pathohistological presentations and different associated lymphoproliferative disorders. Biopsy should rapidly be considered, as early diagnosis of MGRS is essential to initiate clone-directed therapy promptly to prevent progression to ESRD or hematologic progression to malignancy.

## 1. Introduction

Renal impairment caused by the secretion of monoclonal immunoglobulins by clonal B-cells or plasma cells, whether premalignant or nonmalignant, has been recognized. Monoclonal gammopathy of unknown significance (MGUS) is defined by less than 30 g/L of paraprotein or monoclonal immunoglobulin secreted by the plasma cell, less than 10% of bone marrow plasma cell population, and the lack of end-organ damage [1–6]. The term monoclonal gammopathy of clinical significance (MGCS) is used to designate patients who would, in other respect, have MGUS but also have organ damage directly related to the immunoglobulin secretion. Some of these disorders, such as AL amyloidosis, impact many organs including the kidneys [7].

In 2012, the International Kidney and Monoclonal Gammopathy Research Group suggested the term monoclonal gammopathy of renal significance (MGRS) [8] to describe a subset of patients with renal impairment related to the secretion of immunoglobulins without hematological criteria for treatment of a specific disease [5, 9, 10]. In 2017, the definition of MGRS was updated to also include B-cell lymphoproliferative disorders and plasma cell dyscrasias not meeting the criteria for hematologic treatment as causes of MGRS. Thus, this new definition includes certain chronic lymphocytic leukemia (CLL), non-Hodgkin lymphoma, indolent Waldenström macroglobulinemia, and smouldering multiple myeloma [10]. Thus, under the right clinical context, patients with renal impairment and abnormal serum protein electrophoresis should raise the suspicion for MGRS.

Diagnosis of MGRS requires kidney biopsy. Immunofluorescence on biopsy may reveal the presence of monoclonal immunoglobulin deposits within the renal parenchyma [2, 8, 11]. After performing light chain quantification and protein electrophoresis, immunofixation must be done to distinguish between monoclonal immunoglobulin band and other unusual distributions [4–6, 11]. If polyclonal hypergammaglobulinemia is found, a different approach is mandated to elucidate the cause of kidney injury [12]. On the other hand, if a monoclonal band is present, identifying the paraprotein secreting clone must be done with bone marrow aspiration and biopsy. When no clone is identified or when IgM secretion is detected, CT scan or PET scan must be done to identify a suspicious plasmacytoma or adenopathy [2, 5, 8].

**Figure 1:**
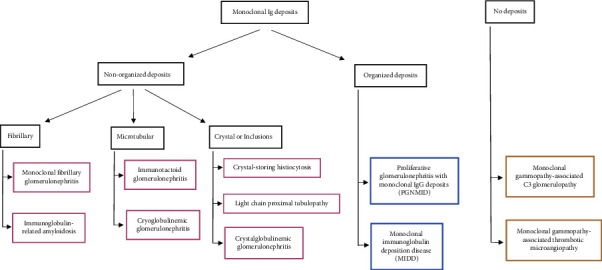
Classification of MGRS. Adapted from Leung et al. [13].

We may classify MGRS according to its histopathology (Figure 1). Renal lesions can be classified into organized or nonorganized immunoglobulin deposits, or nonimmunoglobulin lesions [14, 15]. We present 3 cases of MGRS identified at our center that were either rare or difficult to diagnose.

## 2. Case Presentations

### 2.1. Case 1

The first patient was a woman in her 80s, evaluated in nephrology for nephritic syndrome. The patient was known for CLL for about 10 years, without treatment requirement (Rai stage 0). A gradual increase in creatinine was noted, reaching 211 *μ*mol/L (DFG 18.3 mL/min/1.73 m2) when consulted by a nephrologist (Table 1). Urinalysis revealed the presence of albuminuria, as well as 21 to 50 red blood cells per field and a few leucocytes. The protein/creatinine ratio was 0.99 g/g. The patient then presented with new-onset hypertension. Serum protein electrophoresis revealed an IgG/lambda peak of less than 1 g/L, stable over the last few years. Kappa and lambda light chains were measured at 30.3 mg/L and 103.3 mg/L, respectively, giving a ratio of 0.29, which was considered low for patients with kidney impairment. Bone marrow biopsy demonstrated monoclonal lymphocytosis with lambda+ and CD10+ phenotype. Antibody testing, complement levels, and infectious workup were all negative.

**Figure 2:**
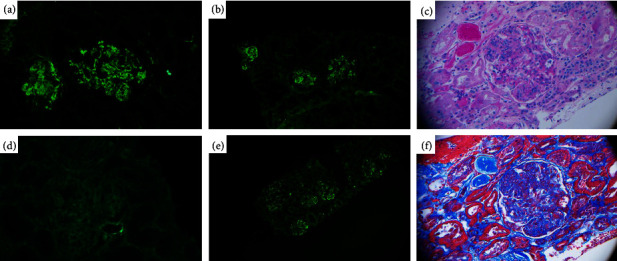
Case 1: pathology slides. (a) Immunofluorescence (IF) microscopy demonstrates C3 granular mesangial and subendothelial deposits of high intensity. (b) IF demonstrates IgG deposits with the same pattern as C3 deposits. (c) Light microscopy shows the PAS membranoproliferative pattern of injury. Nodular mesangial expansion, endocapillary proliferation, subendothelial deposits, and glomerular basement membrane double contour are noted. (d) IF fixation of kappa light chain was negative. (e) IF demonstrates lambda deposits with the same pattern as C3 deposits. (f) Light microscopy Masson's trichrome stain shows the membranoproliferative pattern of injury.

A renal biopsy was done (Figure 2), confirming a diagnosis of monoclonal membranoproliferative glomerulonephritis. Immunofluorescence was notable for the presence of mesangial and subendothelial lambda light chains as well as granular IgG and C3 deposits of various sizes. Congo red staining was negative, and bone marrow biopsy was unremarkable. The patient received 6 cycles of rituximab and chlorambucil (former standard treatment for CLL) for the treatment of MGRS, with creatinine improvement and stabilisation around 100–140. The serum protein electrophoresis remained stable after treatment with an IgG/lambda peak of less than 1 g/L.

### 2.2. Case 2

The second patient was a woman in her 80s who was admitted for rapidly progressive acute renal failure in the context of nephritic syndrome and thrombotic microangiopathy. The creatinine level was at 184 *μ*mol/L (DFG 21.3 ml/min/1.73 m^2^) at admission (Table 1) from a baseline of 50 *μ*mol/L and increased to 287 *μ*mol/L (DFG 21.3 ml/min/1.73 m^2^) the following day. With the bicarbonate level at 10 mmol/L and potassium at 5.1 mmol/L in an oliguric patient, hemodialysis was started immediately. Serum protein electrophoresis demonstrated IgM/kappa paraprotein at 1.8 g/L. Serum kappa and lambda light chains were measured at 79 mg/L and 14 mg/L, respectively, with a ratio of 5.48. PET scan was negative, and bone marrow biopsy showed no evidence of malignancy. Thus, the patient was diagnosed with IgM MGUS.

The protein/creatinine ratio (PCR) was at 2.27 g/g, while the albumin/creatinine ratio (ACR) was at 2.4 mg/mmol. The discordant results between the PCR and ACR were explained by the presence of urine light chains. Urinalysis showed variable count of red blood cells. Finally, thrombocytopenia and hemolytic anemia with schistocytes were noted on blood smear. ADAMTS13 activity was calculated at 22%, thus eliminating thrombotic thrombocytopenic purpura. Serum cryoglobulins measured at three different intervals all came back negative.

**Figure 3:**
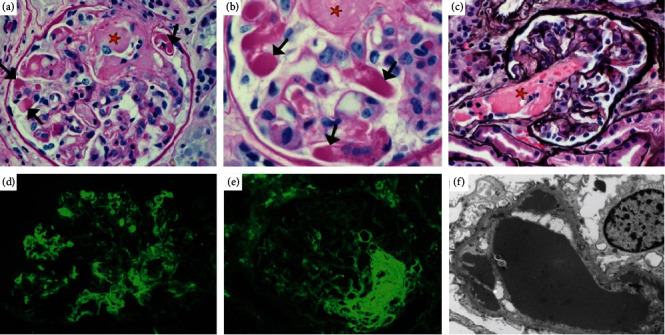
Case 2: pathology slides. (a) Light microscopy shows numerous PAS-positive hyaline pseudothrombi (arrows) as well as a fibrin thrombus (star) (PAS 400x). (b) PAS-positive hyaline pseudothrombi filling the glomerular capillary lumen (PAS 600x). (c) Fibrin thrombosis at the glomerular vascular pole (Jones 200x). (d) Immunofluorescence microscopy demonstrates intracapillary and mesangial staining for kappa light chain. Intracapillary fixation was also noted for IgM, C3, and C1q but not for the lambda light chain. (e) Immunofluorescence microscopy shows fixation of fibrinogen in the glomerular vascular pole, compatible with the thrombosis. (f) Electron microscopy shows unorganized immune-type deposits of intracapillary and subendothelial localisation. Intracapillary fibrin deposits were also noted.

Renal biopsy demonstrated endocapillary proliferative glomerulonephritis associated with the presence of numerous monotypic IgM/kappa intracapillary pseudothrombi (Figure 3). Characteristic changes of thrombotic microangiopathy were also described, with multiple fibrinous intracapillary thrombi. Congo red staining was negative. These findings were compatible with a diagnosis of monoclonal intracapillary deposits and thrombotic microangiopathy related to MGRS in the context of IgM MGUS.

Empiric steroid therapy was started before biopsy results, given the severity of initial presentation with a need for hemodialysis. The patient received Solu-Medrol 1 g daily for 3  days, followed by prednisone 60 mg daily. After obtaining biopsy results, plasmapheresis was initiated and hemodialysis was subsequently rapidly discontinued. Treatment with plasmapheresis was continued for 4  months, after which a deterioration of the renal function was noted. Rituximab was thus started at 375 mg/m^2^ for 4 doses. Two months later, the patient was completely weaned off from prednisone. A follow-up is done a few months later, creatinine was stable at around 110, and there was no evidence of proteinuria. Furthermore, hemolytic parameters were normal and there was no thrombocytopenia. However, the serum protein electrophoresis continued to demonstrate an IgM/kappa paraprotein at 1.1 g/L. The serum kappa light chains remained elevated at 156 mg/L.

### 2.3. Case 3

The third patient was a woman in her 60s who was admitted for progressive renal failure, with an increase in the creatinine level from 66 *μ*mol/L (DFG 72.6 ml/min/1.73 m2) to 117 *μ*mol/L (DFG 42 ml/min/1.73m^2^) a year later (Table 1). Urinalysis revealed the presence of 51 to 100 red blood cells per field, with a protein/creatinine ratio of 1.28 g/g. IgM/kappa paraprotein was also noted to be less than 1 g/L on electrophoresis. Serum light chains kappa and lambda were at 77 mg/L and 10 mg/L, respectively, with an elevated ratio of 7.09.

Suspecting paraprotein-related renal dysfunction, bone marrow biopsy was attempted, but the tissue sample was insufficient for the analysis. The patient was started on empiric treatment with prednisone 60 mg daily.

Renal biopsy was done; however, the specimen quality was limited. Thus, immunofluorescence was impossible to perform, but Congo red staining was negative. With little information from the biopsy, autoimmune glomerulonephritis was erroneously diagnosed. Treatment with CellCept was started, which was changed to Myfortic before completely halting treatment about two years later as the renal function was stable.

Two months later, the creatinine level rapidly rose from 130–150 *μ*mol/L (DFG 30 to 40 ml/min/1.73 m^2^) to 180 *μ*mol/L (DFG 24.6 ml/min/1.73 m^2^). Myfortic was restarted. However, the creatinine level continued to rise to 323 *μ*mol/L (DFG 12.1 ml/min/1.73 m^2^) a few months later. Renal biopsy was repeated. This time, a focally proliferative glomerulonephritis was described. Important tubulointerstitial lesions with fibrosis and atrophy, as well as granular glomerular and tubular IgG, IgM, C3, C1Q, and kappa deposits, were noted. Glomeruli were not permeable for electron microscopy. However, revision of electron microscopy from the first biopsy showed the presence of granular deposits resembling microtubules along the epithelial surface of the glomerular basement membrane. The diagnosis of immunotactoid glomerulonephritis was made. Bone marrow biopsy was repeated. A B-cell infiltrate and monotypic IgM/kappa plasma cells were suspected, without being able to establish a clear diagnosis due to poor quality of the specimen. Thus, conservative management was decided.

Around the same time as the kidney function started to deteriorate, a breast lesion was noted. Biopsy of the breast mass was compatible with diffuse large B-cell lymphoma (DLBCL). Lymphomatous cells expressed IgM/kappa, thus confirming paraprotein-associated renal lesion. The patient received rituximab, cyclophosphamide, doxorubicin, vincristine, and prednisone (R-CHOP) for 6 cycles; however, the kidney function did not recover and dialysis was initiated. The IgM/kappa paraprotein remained stable on electrophoresis. Serum light chains kappa remained elevated.

## 3. Discussion

We described 3 different cases of MGRS, highlighting the diversity of renal pathohistological presentations and different associated lymphoproliferative disorders. The first patient presented with monoclonal membranoproliferative glomerulonephritis in the context of CLL. The second patient had TMA renal lesions from IgM MGUS, which is a very rare MGRS presentation. Finally, the third patient presented with immunotactoid glomerulonephritis likely from small B-cell lymphoma that later transformed to DLBCL.

Despite renal impairment from secreted paraprotein, none of these patients had criteria for treatment of their hematological disease. Indeed, in the first case, the patient's CLL did not warrant treatment with a Rai staging of 0 [16], yet it was likely the cause of the patient's glomerulonephritis, although it is not excluded that the patient could also have a second hematological disease, such as MGUS, that could be the cause of the paraprotein-related renal lesions. Published reports of renal impairment in the context of CLL often highlight the association between the renal dysfunction and paraprotein secreted by B-cell clones [17, 18], with monoclonal membranoproliferative glomerulonephritis being the most common renal presentation [18–21].

The second patient presented with renal TMA lesions in the context of IgM monoclonal gammopathy. Although monoclonal intracapillary deposits and renal TMA are well described with Waldenström macroglobulinemia [22–30], they are however less described with IgM secreting MGUS. A recent retrospective study documented an important prevalence of MGUS in patients with thrombotic microangiopathy, suggesting monoclonal immunoglobulin as a triggering factor [29]. Others have reported associations between these two entities, with the latest consensus from the International Kidney and Monoclonal Gammopathy Research Group adding thrombotic microangiopathy as a provisional status to its classification of lesions associated with MGRS [13]. TMA is not associated with direct paraprotein deposition in the renal parenchyma but rather presents with disordered complement regulation caused indirectly by paraprotein [31]. TMA may be kidney-limited, which highlights the importance of doing a kidney biopsy. Secondary causes of TMA, including pregnancy, infection, drug, autoimmune, transplant, and malignancy, should be evaluated. Genetic analysis for mutations in the complement alternative pathway should be obtained [31], although this was not done in our patient as genetic analyses were not easily accessible.

Finally, the third patient presented with immunotactoid glomerulonephritis, which is a rare cause of MGRS [13, 32]. It is interesting to note that the diagnosis of DLBCL was made a few months after the diagnosis of immunotactoid glomerulonephritis. It is thus most likely that the patient first developed a small lymphocytic lymphoma, which triggered a renal impairment (MGRS), before transforming to DLBCL. Indeed, the presence of the monoclonal IgM/kappa gammopathy on initial electrophoresis, later findings of a B-cell infiltrate with monoclonal IgM/kappa (without classification) on bone marrow biopsy, and finally the diagnosis of DLBCL with similar IgM/kappa expression, all support this hypothesis which triggered a renal impairment (MGRS) before transforming to DLBCL. Associations between immunotactoid glomerulonephritis and CLL or indolent lymphoproliferative processes are well described [33, 34], whereas only one case report of immunotactoid glomerulonephritis in the context of DLBCL was found [35].

As MGRS was only first described in 2012, the true incidence is unknown. In patients with MGUS who underwent renal biopsy, 38% had MGRS lesions in a Chinese retrospective study [36] and 40% had MGRS lesions in a Mayo Clinic retrospective study from 2013 to 2018 [37]. The diagnosis is often delayed because of the requirement of biopsy for diagnosis. Strong predictors of having MGRS include the presence of an elevated free light chain ratio [36], proteinuria, and hematuria [37]. Thus, patients presenting with these features should rapidly be assessed and considered for kidney biopsy to establish a rapid diagnosis to avoid treatment delay and poorer outcomes. Furthermore, patients with MGUS and either atypical evolution of chronic kidney disease, aged less than 50  years, and eligible for kidney transplant should also be further evaluated for potential kidney transplant. However, there is a high risk of recurrence after kidney transplantation [38–41] if the monoclonal gammopathy is not eliminated. This highlights the importance of treating the underlying hematological disease.

Therefore, although paraprotein secreting B-clone may not meet hematological criteria for treatment, when kidney damage from paraprotein results in MGRS, treatment of the underlying clone is warranted to prevent the progression to end-stage renal disease (ESRD) [14, 15, 31, 42]. Multiple case reports have shown renal improvement with treatment of the underlying clone [43, 44]. Furthermore, as observed with the third patient, patients with MGRS are at higher risk of progressing to the corresponding hematological malignancy [14, 45, 46], which also supports the need for treatment.

Although clone-directed therapy is warranted, there is currently no clear guidelines for treatment of MGRS. Therapy is often based on current chemotherapy regimens used for the treatment of the associated malignant clones, as there are very few studies specifically on treatment of MGRS clones, with most reports based on nonrandomized data.

Thus, many patients with IgG, IgA, and light chain plasma cell dyscrasia (non-IgM) are treated with bortezomib [15, 47–49] as a single agent or in combination with other drugs. Studies on proteasome inhibitors are however all retrospective, with no randomized controlled trial specifically for treatment of MGRS. However, in multiple myeloma, bortezomib-containing regimens have shown to reduce tumor load and improve the kidney function [50–52].

The first patient in our case series with CLL was treated with rituximab and chlorambucil. In a retrospective study from 1975 to 2014 at the Mayo Clinic [21], all five patients with CLL complicated by MGRS treated with rituximab, cyclophosphamide, and steroid (RCP-based) regimen had recovery of the renal function, as opposed to none of the three patients with rituximab with or without steroids. However, since then, treatment for CLL has shifted to tyrosine kinase inhibitors, with now case reports of patients receiving ibrutinib in combination of rituximab-based regimens for treatment of MGRS [53]. It is however still unclear what the optimal treatment for paraprotein-associated renal lesions in CLL is.

The second patient had renal TMA in the context of IgM MGUS. As this is a rare hematological entity with a rare renal presentation, there is little evidence for treatment of MGRS-related IgM paraprotein. Rituximab-based therapy, as received by our patient, can be considered [42] as it is usually the treatment of choice for the associated malignancy of Waldenström macroglobulinemia.

Finally, a B-cell clone could only be identified in the third patient after progression from an indolent lymphoproliferative process to DLBCL. Thus, the patient received R-CHOP. However, ideally, the patient should have been treated for MGRS before progression to DLBCL, although the optimal treatment regimen would have been unclear as there are no specific treatment guidelines for MGRS clone-directed therapy.

Optimal timing for starting chemotherapy is also unknown in patients with MGRS. Treatment is also challenged by the lack of MGRS response criteria. Studies [47, 48, 54] are based either on multiple myeloma response criteria [55], or immunoglobulin light chain amyloidosis treatment response criteria [56], as amyloidosis is a common MGRS presentation [36, 37]. However, there is no renal response criterion for either of these consensuses, as it was not designed with MGRS in mind. Thus, development of MGRS-specific response criteria is called for, as treatment is warranted mostly for prevention of progression to ESRD.

Rapid management and multidisciplinary approach are essential [15]. In previous series, kidney biopsies were deferred in approximately 40% of cases because of lack of awareness of kidney disease by the hematologist, or lack of awareness of MGUS by the nephrologist [37]. It is therefore important to increase physician awareness of MGRS and to encourage physicians to search for the association between renal disease and monoclonal gammopathy. Finally, our work highlights the importance of the nephrologist's and hematologist's cooperation for early diagnosis and optimal care management.

## 4. Conclusion

We present 3 singular cases of MGRS from our institution. These cases highlight the diversity of renal histopathological presentation and associated hematological disorder. Biopsy should rapidly be considered in patients with renal impairment and any B-cell lymphoproliferative disorders or plasma cell dyscrasia not meeting hematological criteria for treatment. Early diagnosis of MGRS is essential to initiate clone-directed therapy promptly to prevent progression to ESRD or hematologic progression to malignancy. Finally, we hope for a greater recognition of MGRS and for the elaboration of eventual randomized studies for treatment.

## Figures and Tables

**Table 1:** Patient laboratory results at presentation.

	Patient 1	Patient 2	Patient 3
Serum creatinine (*μ*mol/L)	211	184	117
DFG (mL/min/1.73 m^2^)	18.3	21.3	42
Urine albumin/creatinine ratio (0–1.9 mg/mmol)	—	2.4	
Urine protein/creatinine ratio (0–0.2 g/g)	0.99	2.27	1.28
Serum protein electrophoresis	IgG/lambda peak <1 g/L	IgM/kappa 1.8 g/L	IgM/kappa <1 g/L
Serum-free light chains (mg/L)	Kappa 30.3	Kappa 79	Kappa 77
Kappa (3.3–19.4 mg/L)	Lambda 103.3	Lambda 14	Lambda 10
Lambda (5.7–26.3 mg/L)	Ratio 0.29	Ratio 5.48	Ratio 7.09
Bence Jones	Lambda	Kappa	Kappa
Urine protein electrophoresis	Nonselective glomerular proteinuria	Tubular proteinuria	

*Total serum calcium*			
C3 (0.90–1.80 g/L)	N	0.43	N
C4 (0.10–0.45 g/L)	N	0.08	<0.05
Antineutrophil cytoplasmic antibody (ANCA)	Negative	Negative	—
Antiglomerular basement membrane antibody (anti-GBM)	Negative	Negative	—
Antinuclear factor	Negative	—	Negative
Rheumatoid factor	Negative	Negative	Negative
Hepatitis B and C serologies	Negative	Negative	Negative
Serum cryoglobulins	Negative	—	Negative
Red Congo staining on biopsy	Negative	Negative	Negative
C-reactive protein (0–8 mg/L)	5.3	39.1	100
IgG (7–16 g/L)	5.48	5.10	1.6
IgM (0.7–4 g/L)	0.29	16.4	7.02
IgA (0.4–2.30 g/L)	0.70	0.68	0.17

## Data Availability

Due to patient confidentiality issues, data will not be available.
